# Outcomes and outcome measures used in evaluation of communication training in oncology – a systematic literature review, an expert workshop, and recommendations for future research

**DOI:** 10.1186/s12885-019-6022-5

**Published:** 2019-08-14

**Authors:** F. Fischer, S. Helmer, A. Rogge, J. I. Arraras, A. Buchholz, A. Hannawa, M. Horneber, A. Kiss, M. Rose, W. Söllner, B. Stein, J. Weis, P. Schofield, C. M. Witt

**Affiliations:** 1 Department of Psychosomatic Medicine, Center for Internal Medicine and Dermatology, Charité – Universitätsmedizin Berlin, corporate member of Freie Universität Berlin, Humboldt-Universität zu Berlin, and Berlin Institute of Health, Berlin, Germany; 2 Institute for Social Medicine, Epidemiology and Health Economics, Charité – Universitätsmedizin Berlin, corporate member of Freie Universität Berlin, Humboldt-Universität zu Berlin, and Berlin Institute of Health, Berlin, Germany; 3 Radiotherapeutic Oncology Department & Medical Oncology Department, Complejo Hospitalario de Navarra, Pamplona, Spain; 4 Department of Medical Psychology, Centre for Psychosocial Medicine, University Medical Centre, Hamburg, Germany; 5 Center for the Advancement of Healthcare Quality and Patient Safety (CAHQS), Faculty of Communication Sciences, Università della Svizzera Italiana, Lugano, Switzerland; 6 Department of Internal Medicine, Divisions of Pneumology and Oncology/Hematology, Paracelsus Medical University, Klinikum Nuernberg, Nuernberg, Germany; 7 Department of Psychosomatic Medicine, University Hospital Basel, Basel, Switzerland; 8 Department of Quantitative Health Sciences, Outcomes Measurement Science, University of Massachusetts Medical School, Worcester, USA; 9 Department of Psychosomatic Medicine and Psychotherapy, Paracelsus Medical University, Nuremberg General Hospital, Nuremberg, Germany; 10 Comprehensive Cancer Center, Department of Self-Help Research, Faculty of Medicine and Medical Center University of Freiburg, Freiburg, Germany; 11 Department of Psychology, Swinburne University, Melbourne, Victoria Australia; 12 Department of Cancer Experiences Research, Peter MacCallum Cancer Centre, Melbourne, Victoria Australia; 13 Sir Peter MacCallum Department of Oncology, The University of Melbourne, Parkville, Victoria Australia; 14 Institute for Complementary and Integrative Medicine, University Hospital Zurich and University of Zurich, Zurich, Switzerland; 15 Center for Integrative Medicine, University of Maryland School of Medicine, Baltimore, MD USA

**Keywords:** Communication training, Evaluation, Oncology, Outcome

## Abstract

**Background:**

Communication between health care provider and patients in oncology presents challenges. Communication skills training have been frequently developed to address those. Given the complexity of communication training, the choice of outcomes and outcome measures to assess its effectiveness is important. The aim of this paper is to 1) perform a systematic review on outcomes and outcome measures used in evaluations of communication training, 2) discuss specific challenges and 3) provide recommendations for the selection of outcomes in future studies.

**Methods:**

To identify studies and reviews reporting on the evaluation of communication training for health care professionals in oncology, we searched seven databases (Ovid MEDLINE, CENTRAL, CINAHL, EMBASE, PsychINFO, PsychARTICLES and Web of Science). We extracted outcomes assessed and the respective assessment methods. We held a two-day workshop with experts (*n* = 16) in communication theory, development and evaluation of generic or cancer-specific communication training and/or outcome measure development to identify and address challenges in the evaluation of communication training in oncology. After the workshop, participants contributed to the development of recommendations addressing those challenges.

**Results:**

Out of 2181 references, we included 96 publications (33 RCTs, 2 RCT protocols, 4 controlled trials, 36 uncontrolled studies, 21 reviews) in the review. Most frequently used outcomes were participants’ training evaluation, their communication confidence, observed communication skills and patients’ overall satisfaction and anxiety. Outcomes were assessed using questionnaires for participants (57.3%), patients (36.0%) and observations of real (34.7%) and simulated (30.7%) patient encounters. Outcomes and outcome measures varied widely across studies. Experts agreed that outcomes need to be precisely defined and linked with explicit learning objectives of the training. Furthermore, outcomes should be assessed as broadly as possible on different levels (health care professional, patient and interaction level).

**Conclusions:**

Measuring the effects of training programmes aimed at improving health care professionals’ communication skills presents considerable challenges. Outcomes as well as outcome measures differ widely across studies. We recommended to link outcome assessment to specific learning objectives and to assess outcomes as broadly as possible.

## Background

Communicating with cancer patients, for example disclosing the diagnosis, discussing treatment and providing emotional support in discussions about end of life, can be challenging: [1]. Hence, effective communication skills are considered vital to high quality cancer care [2]. Programmes have been developed and conducted to train physicians and other health care professionals (HCPs) to communicate more effectively with cancer patients [3, 4]. Although intuitively appealing, a recent review of randomized controlled trials investigating the benefit of communication skill training (CST) showed mixed results. While an improvement in HCPs’ communication skills was reported for some programmes, effects on patient-reported outcomes, such as psychological distress or quality of life, have not been established yet [5]. This was also reported in earlier reviews [6, 7]. Nonetheless, experts agree that the ultimate objective of clinician-patient communication training is to improve patient outcomes, such as adherence, self-efficacy health-related quality of life [6].

The choice of appropriate outcomes and the instrument to measure these (outcome measures) is critical to accurately assess the effectiveness of CST [8, 9]. It has been demanded to closely link outcomes with the content of the CST, to use only validated scales as outcome measures and to assess long-term effects of the intervention [10]. This can be challenging as outcomes directly linked to an intervention (proximal outcomes) might be considered less relevant as distal outcomes, particularly for long-term follow-up [11], and validated scales are sparse for narrowly defined outcomes. Eventually, many different outcome measures have been developed and used in the past, and as a result, there are no standards for appropriate evaluation (i.e., methodology and measurement) of clinician-patient communication training in oncology.

Therefore, this paper aims to

1. Provide an overview of the outcomes and outcome measures as well as the respective assessment methods used for CST in oncology,
2. Identify challenges that have been encountered in the evaluation of CST in oncology,
3. Provide recommendations to address these challenges in future research.

To achieve these aims, we 1) performed a systematic review of the literature and identified outcomes and outcome measures that have been used to evaluate the effects of CST, 2) convened a workshop involving international experts to discuss challenges in assessing outcomes of CSTs to complement the review and 3) developed recommendations to address these challenges in future evaluations of CSTs.

## Methods

### Systematic review

We conducted a systematic review to identify outcomes assessed as well as the respective outcome measures used in the field. We specified a protocol, which is available at https://tinyurl.com/yd5hyggt. We searched seven electronic databases (Ovid MEDLINE, CENTRAL, CINAHL, EMBASE, PsychINFO, PsychARTICLES and Web of Science) in December 2016 for publications reporting on the effects of standardized CST in oncology. In addition, we hand-searched reference lists of the 21 identified reviews for relevant studies missed by our search.

We combined search terms describing aspects of physician-patient relations that are common goals of CST (communication, empathy, interaction, …) with terms describing structured programmes (course, curriculum, training, …). Search terms were informed by previous reviews [4, 5, 8, 9, 12], which mainly investigated the effects of standardized communication trainings. We used MeSH terms and limits to restrict the results to trials and observational studies in adult cancer patients, depending on the respective database. Explicit search terms are listed in Table 1.

**Table 1:** Search terms for MEDLINE search

Search terms	Limiters
(((AB (communicat* OR empath* OR ‘interaction’ OR ‘interpersonal’ OR ‘interview’ OR ‘patient relation’ OR ‘shared decision making’) OR TI (communicat* OR empath* OR ‘interaction’ OR ‘interpersonal’ OR ‘interview’ OR ‘patient relation’ OR ‘shared decision making’))AND (AB (teach* OR session OR educat* OR program* OR instruction OR curriculum OR course OR training OR workshop OR skills) OR TI (teach* OR session OR educat* OR program* OR instruction OR curriculum OR course OR training OR workshop OR skills)) AND (AB (evaluation OR assessment OR effects OR study OR trial OR investigation) OR TI (evaluation OR assessment OR effects OR study OR trial OR investigation)))) AND MM “Neoplasms”	Abstract Available; Human; Age Related: Young Adult: 19–24 years, Adult: 19–44 years, Middle Aged: 45–64 years, Middle Aged + Aged: 45 + years, Aged: 65+ years, Aged, 80 and over, All Adult: 19+ years; Subject Subset: Cancer; Publication Type: Clinical Trial, Clinical Trial, Phase I, Clinical Trial, Phase II, Clinical Trial, Phase III, Clinical Trial, Phase IV, Comparative Study, Controlled Clinical Trial, Evaluation Studies, Meta-Analysis, Multicenter Study, Randomized Controlled Trial, Review, Validation Studies; Language: English, German

Inclusion criteria were interventional or observational studies or reviews, which assessed the effects or evaluated standardized CST tailored to physicians and/or other health care professionals focusing on communication with adult cancer patients. In addition, these needed to be published in a scientific outlet or as publicly available reports, working papers or theses. Publications were excluded if the outcome assessment was not standardized in the specific study, e.g., not all participants were evaluated using the same method, or if the publication was available in neither English nor German.

One reviewer (FF) checked all references found in the literature search and excluded clearly irrelevant articles based on titles and abstracts. We obtained full text copies from all remaining articles and two reviewers (FF, AR) assessed those independently for eligibility. We assessed the agreement of their selections by calculating the kappa statistic. We excluded publications when both reviewers agreed. We documented reasons for exclusion and resolved disagreements by discussion. If several reports for a single study were identified, all publications were reviewed for eligibility.

We grouped outcome measures in original research into the respective underlying constructs, and counted the frequency of their use. Along with information about the outcomes assessed, we extracted the study design, sample size, target group and intervention characteristics. As the results of the included studies were not of interest, we did not assess the risk of bias.

As reviews on the efficacy of CST potentially contained relevant information about challenges in outcome choice and outcome measurement, we included them in our review. We extracted and qualitatively synthesized arguments regarding outcomes and the respective outcome measures. To avoid redundancy, we did not extract information about outcomes and outcome measures used in primary data from the reviews.

In general, we followed the PRISMA reporting guidelines [13], although some items were not applicable given the scope of the review.

### Expert workshop

We held a two-day workshop in Berlin, Germany in February 2017. The aim of the workshop was to complement the systematic review by identifying challenges in the evaluation of communication training in oncology and to discuss ways to address those challenges in future research.

We invited researchers from the “Kompetenznetzwerk Komplementärmedizin in der Onkologie” KOKON, who investigate communication about complementary medicine, to the workshop. We also defined fields for which we sought additional expertise. These fields were communication theory, development and evaluation of generic or cancer-specific communication training and/or outcome measure development. Experts in these fields were identified based on their occurance in the review as well as through suggestions by other invited researchers. Overall, 16 experts, including a patient representative, took part in the workshop (see Table 2).

**Table 2:** Participants in the expert workshop

Participant	Affiliation	Country
Juan Ignacio Arraras	Complejo Hospitalario de Navarra, Radiotherapeutic Oncology Department & Medical Oncology Department, Pamplona	Spain
Angela Buchholz	Department of Medical Psychology, University Medical Center Hamburg-Eppendorf	Germany
Felix Fischer	Department of Psychosomatic Medicine, Center for Internal Medicine and Dermatology, Charité – Universitätsmedizin Berlin	Germany
Corina Güthlin	Institute of General Practice, Johann Wolfgang Goethe University, Frankfurt/Main	Germany
Stefanie Helmer	Institute for Social Medicine, Epidemiology and Health Economics, Charité – Universitätsmedizin Berlin	Germany
Annegret Hannawa	Center for the Advancement of Healthcare Quality and Patient Safety (CAHQS), Faculty of Communication Sciences, Università della Svizzera Italiana, Lugano	Switzerland
Markus Horneber	Department of Internal Medicine, Divisions of Pneumology and Oncology/Hematology, Paracelsus Medical University, Klinikum Nuernberg	Germany
Ulrike Holtkamp	German Leukemia & Lymphoma Patients’ Association	Germany
Alexander Kiss	Department of Psychosomatic Medicine, University Hospital Basel	Switzerland
Christin Kohrs	Department of Internal Medicine, Division of Oncology and Hematology, Paracelsus Medical University, Klinikum Nuernberg	Germany
Darius Razavi	Psychosomatic and Psycho-Oncology Resarch Unit, Université Libre de Bruxelles, Brussels	Belgium
Matthias Rose	Department of Psychosomatic Medicine, Center for Internal Medicine and Dermatology, Charité – Universitätsmedizin Berlin	Germany
Jan Schildmann	Institute for History and Ethics of Medicine, Martin Luther University Halle-Wittenberg	Germany
Penelope Schofield	Department of Psychology, Swinburne University, Melbourne	Australia
Barbara Stein	Department of Internal Medicine, Division of Oncology and Hematology, Paracelsus Medical University, Klinikum Nuernberg	Germany
Claudia Witt	Institute for Complementary and Integrative Medicine, University Hospital Zurich and University of Zurich	Switzerland

We organised the workshop into four parts:

1. Participants shared their perspectives and experiences regarding development and evaluation of communication trainings. In this part, we posed four broad questions: (a) what are good practices when communicating with oncology patients, (b) what are the desirable effects of good communication, (c) how one can generally assess quality of communication, and (d) what are experiences from evaluations of CST. Additionally, we presented preliminary results of the review. Participants wrote Issues elicited that were important for a valid assessment/evaluation of CST on cards.
2. The participants then clustered those cards on a board into broader topics to identify areas that needed to be considered when measuring the effects of CST. Then, we identified three main topics for further discussion.
3. The members participated in structured, small group discussions focusing on the three topics. We assigned participants to one of the three groups. Each group discussed one of the three topics for 20 min prior to rotating to the next group. Three ‘discussion leaders’ were each assigned to one of the three topics to guide the small group discussion.
4. Discussion leaders presented the results obtained in step 3 to the entire group, and we discussed these results in a plenary session.

### Development of expert recommendations

After the workshop, we drafted recommendations for future evaluations of communication training in oncology based on the results of the systematic review as well as the experts’ discussions. We invited workshop participants to comment on the recommendations during manuscript preparation, and the recommendations were adapted until no further comments were made.

## Results

### Systematic review

#### Search results

**Fig. 1:**
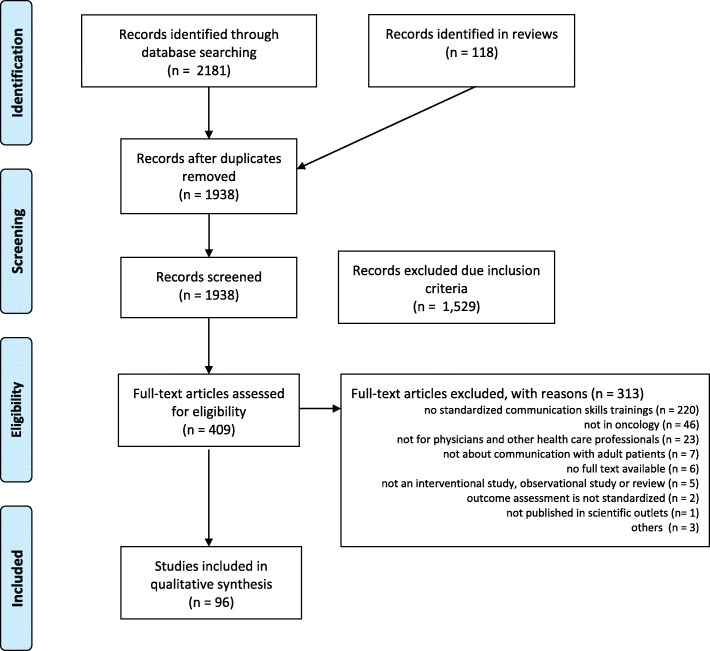
Flowchart for literature search and study selection

Overall, our search retrieved 2181 references. We identified an additional 118 references by examining reference lists in identified reviews on communication training. After removing duplicates, we screened 1938 abstracts and excluded 1529 because they did not fulfill inclusion criteria, leaving 409 references for full text analysis. Of these, 313 publications did not fulfill inclusion criteria and were therefore excluded, leaving 96 publications for inclusion in the review. The agreement on exclusion between reviewers was moderate (kappa = 0.56), with consistent decisions on 351 articles. All conflicts were resolved through discussion. We give the detailed reasons for the exclusion of references in Fig. 1.

#### Included studies

Of the 96 publications found eligible for synthesis, 33 reported on randomized controlled trials (RCTs), 2 were RCT protocols of so far unpublished trials, 4 were controlled trials (group allocation not randomized), 36 were uncontrolled studies and 21 were reviews.

The number of participants included in studies reporting on primary data ranged from 3 to 515, with 50% of studies reporting sample sizes between 30 and 114. The participants of the CST were physicians in 51% of the studies, nurses in 36%, mixed health care providers (mostly physicians and nurses) in 11% and other health care professionals (e.g., speech therapists) in 3%. Out of 33 RCTs, 19 compared participants of a CST with a waiting list control group, 7 compared different forms of CST, e.g., workshops of varying length or by adding consolidation workshops, 6 compared a CST to a no training condition, and in one RCT, it was unclear whether the control group received any intervention. Two of the four controlled trials compared interventions with a waiting list, whereas 1 compared a basic with an extended intervention, and 1 study compared performance of the same sample before and after completing the intervention. In the uncontrolled studies, 33 of 36 followed a pre-post design, comparing outcomes before and after the intervention, while 3 assessed outcomes only after the intervention.

#### Overview of outcomes

The articles reporting primary data and study protocols reported on average 3.2 (sd = 2.2, range = 1–10) distinct outcome measures. 43 (57.3%) articles reported outcome data collected from CST programme participants, 27 (36.0%) from patients of the programme participants, 26 (34.7%) reported on observations of real and 23 (30.7%) on simulated communication encounters, and 9 (12%) reported on other types of outcome measures. Approximately half of the studies (37/49.3%) reported data from one of these sources only, one-third (25/33.3%) two sources, 11 (14.7%) three sources and 2 (2.7%) four sources.

#### CST participant questionnaires

Overall, 43 studies (11 RCTs, 2 RCT protocols, and 25 trials/observational studies) reported 93 outcomes collected with questionnaires for CST participants. The most frequently reported data were from training evaluation questionnaires, followed by questionnaires obtaining self-ratings on aspects of the respondents’ communication (communication confidence (16), communication self-effectiveness (4), communication skills (3), communication practice (1)) and respondents’ distress/burnout (16). The outcomes and the respective instruments are listed in Table 3.

**Table 3:** Outcomes and respective measures for the assessment of training participants

Outcome construct	Outcome measure	Number of studies	References
Training evaluation	purpose built	25	[14–38]
Communication confidence	Baile’s Questionnaire [39]	16	[20, 22, 39, 40]
	Fallowfield’s Questionnaire [30]		[30, 41, 42]
	modified Communication Outcomes Questionnaire [43]		[39]
	purpose built		[16, 23, 26, 28, 30, 33, 40, 44–46]
Distress	General Health Questionnaire [47]	16	[21, 22]
	Maslach Burnout Inventory [48]		[17, 19, 20, 22, 35, 49, 50]
	Nursing Stress Scale [51]		[35, 50, 52, 53]
	purpose built		[54, 55]
Communication self- effectiveness	modified Communication Outcomes Questionnaire [43]	4	[56–58]
	purpose built		[50]
Attitudes towards cancer	Physician Psychosocial Belief Scale [59]	3	[46]
	purpose built using a semantic differential [60]		[52, 53]
Communication skills	modified Nurses’ Basic Communication Skills Scale [61]	3	[58]
	Perception of the Interview Questionnaire [62]		[52]
	purpose built		[33]
Implementation of training elements in practice	purpose built	3	[28, 29, 46]
Expectations on the consultation	modified Communication Outcomes Questionnaire [43]	3	[39, 58]
	purpose built		[16]
Satisfaction with consultation given	purpose built	3	[16, 52, 56]
Communication practices within the department	purpose built	2	[23, 36]
Anxiety	State-Trait Anxiety Inventory [63]	1	[56]
Attitudes towards caring	Attitudes Towards Caring for Patients Feeling Meaninglessness instrument	1	[34]
Attitudes towards dying	Frommelt Attitude Towards Care of the Dying [64]	1	[34]
Attitudes towards clinician-patient-relationship	Doctor-Patient rating [65]	1	[25]
Confidence in information provision	purpose built [66]	1	[17]
Coping	purpose built	1	[36]
Empathy	Test of Empathic Capacity [67]	1	[25]
Knowledge	purpose built	1	[29]
Patient-centeredness	Words emotionally related to dying test [68]^a^	1	[46]
Perceived support	Nurses’ Self-Perceived Support Scale [69]	1	[58]
Clinician-patient relationship	Nurse-Patient Relationship Inventory [70]^a^	1	[50]
Sense of coherence	Sense of Coherence-13 [71]	1	[34]
Shared decision-making behaviour	Mapping-Q [72]	1	[73]
Social support	purpose built	1	[36]
Truth-telling preference	Truth Telling Questionnaire [74]	1	[75]

^a^reference could not be retrieved

#### Patient questionnaires

A total of 26 studies (18 RCTs, 2 RCT protocols of so far unpublished trials, and 6 trials/observational studies) reported on 84 (35 unique constructs) outcomes collected with questionnaires for patients of CST participants. Most frequently, patients’ overall satisfaction was assessed (12), followed by anxiety (10), generic quality of life (6) and depression (5). All outcomes assessed and the respective instruments are listed in Table 4.

**Table 4:** Outcomes and respective measures for the assessment of patients

Outcome construct	Outcome measure	Number of studies	Studies
Satisfaction	adapted Client Satisfaction Questionnaire [76]	12	[77]
	adapted from Korsch et al. [78]		[14]
	Cancer Diagnostic Interview Scale [79]		[80]
	EORTC Cancer Outpatient Satisfaction with Care Questionnaire [81]		[54]
	Medical Interview Satisfaction Scale [82]		[77, 83]
	Patient Satisfaction Questionnaire III [84]		[85]
	Patient Satisfaction with Communication Questionnaire [86]		[41]
	purpose built		[16, 49, 87, 88]
Anxiety	Hospital Anxiety and Depression Scale [89]	10	[50, 55, 90–92]
	State-Trait Anxiety Inventory [63]		[14, 41, 49, 77, 91]
Quality of life	EORTC Quality of Life Questionnaire (QLQ)-C-30 [93]	6	[49, 50, 87]
	EORTC Quality of Life Questionnaire (QLQ)-C-15 Pal [94]		[85]
	Perceived Adjustment to Chronic Illness Scale [95]		[49]
	8 Item Short Form Health Survey (SF 8) [96]		[87]
Depression	Beck Depression Inventory [97]	5	[77, 80]
	Hospital Anxiety and Depression Scale [89]		[50, 55, 90]
Distress	Brief Symptom Inventory	5	[80]
	General Health Questionnaire [47]		[41]
	Hospital Anxiety and Depression Scale [89]		[40, 98]
	purpose built		[80]
Empathy	Consultation and Relational Empathy Measure [99]	3	[98, 100]
	purpose built		[101]
Knowledge	Ellis Clinical Trials Knowledge [66]	3	[14]
	purpose built		[49, 73]
Information and control preference	(modified) Information & Control Preference Scale [102]	3	[14, 49]
	Quality of Care Through the Patients’ Eyes (QUOTE-gene-CA) [103]		[104]
Satisfaction with decision	Satisfaction with Decision Scale [105]	3	[14, 49, 98]
Communication skills	Perception of the Interview Questionnaire [62]	2	[52]
	purpose built		[85]
Decisional conflict	Decisional Conflict Scale [106]	2	[14, 98]
Clinician-patient relationship	Nurse-Patient Relationship Inventory [107]^a^	2	[50]
	purpose built		[100]
Quality of care	Palliative Care Outcome Scale [108]	2	[85]
	purpose built		[85]
Shared decision-making behaviour	MAPPIN-Q [72]	2	[73]
	Shared Decision Making Questionnaire [109]		[98]
Trust in clinician	purpose built	2	[40, 101]

^a^reference could not be verified

#### Observations of real patient encounters

A total of 26 articles (14 RCTs, 2 RCT protocols, and 10 trials/observational studies) reported on observations of real patient encounters. Outcomes assessed were communication skills, e.g., supportive utterances or eliciting patients’ thoughts [14–16, 52, 54, 55, 83, 87, 90, 91, 101, 104, 110–118], actual content of the interview [41, 42, 104, 116] and shared decision making behaviour [17, 73].

Encounters were either audio-recorded (17), video-recorded (10) or both (1), partly transcribed and rated using mostly self-developed or adapted coding systems. In general, each coding system defines a number of behaviours or utterances, and observers rate their occurrence subsequently. Those behaviours are usually derived from a clearly defined model of communication. For example, the coding system employed by Wilkinson et al. [117] reflects key areas of a nurse interview, and Fukui et al. [55, 87, 90] connects behaviour to the 6 steps of the SPIKES protocol. Only in one paper [113], authors used an established coding scheme without adaption (MIPS [119]). Publications using the same coding systems were mostly from the same research group.

Several measures were usually taken to ensure the quality of the rating process. These included blinding of the raters, rater training and assessment of inter-rater reliability in the full or a subsample of recorded observations or rater supervision by an experienced rater. In two studies, transcripts were automatically coded using specialized software along with context-specific dictionaries [54, 110].

#### Observations of simulated patient encounters

A total of 23 references ([13] RCTs, 10 trials/observational studies) reported on observations of simulated patient encounters. Most studies assessed communication skills [15, 18–21, 40, 44, 52, 53, 56, 57, 80, 88, 110, 120–127]. In two studies, the content of the interview was explicitly assessed as the number of elicited concerns specified in the actors role [19] and observed key aspects from guidelines [44]. The reaction to scripted cues [21] and the working alliance [127] were each assessed in one study.

In 10 cases, encounters were video-taped, whereas in 11 they were audio-taped; in 2, it was unclear whether encounters had been recorded. Similar to observations of real patient encounters, in most cases, (20) self-developed or adapted ratings of communication behaviour were assessed [18–20, 57, 75, 80, 120–122, 126]. The most frequently used rating system was an adaption of the Cancer Research Campaign Workshop Evaluation Manual (CRCWEM) [52, 53, 88, 110, 125, 128]. All these studies were conducted by the same research group. Three studies [40, 123, 127] used adapted versions of the Roter Interaction Inventory, and one study [124] assessed communication behaviour using the Medical Interaction Process System MIPS [119].

#### Other outcomes

A total of 10 outcome measures in 9 studies were assessed using other methods than direct observations of a communication situation or questionnaires for health care professionals or patients. In one case, objective measures (HCPs’ heart rate and cortisol level) were used to measure stress [56]. Another strategy was to use open questions on either case vignettes or actual communication encounters to test knowledge on communication models [21, 22, 129] or interview either patients or programme participants [23, 115]. Additionally, observable patient behaviour, such as uptake of a treatment or screening participation [73] or as feedback from simulation patients [24, 44], served as outcome.

#### Outcome assessments in reviews on the efficacy of CSTs

A total of 21 reviews assessed the efficacy of CST. In 7 of these 21 reviews, the choice of outcome measures in the included studies was not discussed [3, 130–135].

One review commented that the term “communication” was used vaguely and inconsistently across studies [136], and another concluded that studies often did not clearly define which specific communication competencies were addressed by the respective CST [6]. Consequently, these problems hampered the comparability of studies [137]. Hence, it has been suggested that core communication competencies should be defined to guide future research [138], preferably in terms of an overall score with some key dimensions [6]. Such a communication model for a specific domain can be developed, for example, within a meta-synthesis [139]. For example, researchers could identify critical internal and external factors in the domain of breaking bad news that could be used to inform the development of the CST as well as the desired outcome [139]. A key challenge is that it may be impossible to define communication behaviours that are appropriate in all given situations [140].

Outcome assessment must be aligned to the specific aim of the CST [7, 10] with a formal definition of the communication behaviour that is being taught. Some authors argued that a change in patient outcomes is the ultimate goal of communication training [6, 137], but communication training can also be seen as a vital resource for HCPs to reduce work-induced stress [141]. It has been proposed to employ an outcome measurement framework – such as Kirkpatrick’s triangle [137], which differentiates different levels of impact of the training, or a more specific framework detailing the possible effects of a communication training in the context of oncology [142].

Although self-reports of the participants have been frequently obtained, these are more prone to bias compared to more objective measurements, e.g., through observation of communication behaviour [136]. Consequently, the latest, most comprehensive Cochrane review on the effects of communication trainings in oncology specifically excludes self-reported outcomes on knowledge and attitudes as those are prone to optimistic bias [5]. Furthermore, generic outcomes, such as overall satisfaction of patients, have been found to be sensitive to ceiling effects, making it difficult to measure improvement through CST [7]. On the other hand, it has been argued that direct observations of clinical encounters can also be biased as this might be intrusive [143]. Arguably, there is a need to assess patient outcomes more frequently [6, 144] and to investigate the impact of an intervention on the whole medical team [144]. However, existing reviews indicated that the effect of CST on patients is small [5, 7]. It is unclear whether this is because of competing influences on patient outcomes or an inappropriate choice of outcome measures.

The reviews agree that it would be desirable to concentrate on a single pre-specified outcome measure [5, 7, 145] and to use validated scales for outcome assessment [5, 10].

### Results of the workshop

We identified additional challenges in the evaluation of CST during the workshop, which are presented in Table 5.

**Table 5:** Challenges in the choice of outcomes and outcome measures for CSTs in oncology

Challenge	Description
Communication skills and the outcomes of communication encounters between health care professionals and their patients are related to many internal and external variables.	HCPs communication is influenced by trait factors such as extraversion, state variables such as current stress level and work satisfaction as well as personal knowledge. The same is true for patients, who also have different personality factors and information bases as well as emotional needs and may be at different stages in the illness trajectory. A specific communication encounter will be additionally influenced by external factors that shape the communication situation, such as availability of time and its implementation in clinical routine.
It is hard to define ‘correct’ communication behaviour.	HCPs communication styles and patients’ needs addressable by communication differ widely, both across patients and during the course of disease. Communication often takes unpredictable turns and miscommunication is frequent; this does not necessarily imply that the outcome of a miscommunication is bad.
Targeting of CST can be improved.	Highly motivated HCPs with good communication skills are more likely to take part in CSTs than HCPs with bad communication styles. Therefore, ceiling effects, both in actual effects and their measurement, have been frequently observed. Patients’ needs must be adequately addressed in the conceptualization of the training.
Learning objectives of CST vary widely.	CSTs differ widely in their specificity (generic communication training, such as active listening and expressing empathy vs. training tailored to specific communication tasks such as breaking bad news). If a CST is focused on a specific communication task, consideration needs to be given to all the skills required to satisfactorily deal with the situation.
Communication affects many different outcomes.	CSTs target many different outcome parameters. Some of them are closely connected to the content of the CST (proximal outcomes), others are influenced by many other factors as well (distal outcomes). While proximal outcomes are more likely to reflect changes after a CST, there are known problems. For example, measures of satisfaction of CST participants have frequently exhibited ceiling effects. Additionally, empathy was considered an important construct by experts but difficult to measure in an objective way. It seems to be difficult to define the appropriate measurement to capture proximal outcomes, such as clinician skill in expression of empathy. Distal outcomes such as Anxiety, Distress and Quality of Life are influenced by many other factors besides communication and the effect of a communication training on such distal outcomes has often been limited.
Validated measures are not available for specific outcomes of interest.	The limited availability of validated scales for proximal outcomes was identified by experts as a considerable barrier. This also implies that it is unclear what minimal important differences are on such scales. Scales measuring generic, broadly applicable outcomes are more likely to be used and validated. Most outcomes for which validated measures exist are distal. The imperative in research to employ validated scales might influence researchers to select generic outcomes, which may not be optimally aligned with the goals of a particular CST.

Participants identified three main domains of outcomes for further discussion:

1. Outcomes related to the HCP taking part in the CST, such as their communication skills or satisfaction with the training,
2. Outcomes related to a specific interaction between HCPs and patients,
3. Outcomes related to the patients who communicate with the trained HCP.

Overall, experts agreed that a “one size fits all approach” is not appropriate in defining outcomes for CST evaluations; thus, we cannot give recommendation on specific constructs. Rather, outcomes need to be dependent on the specific learning objectives of the CST under evaluation. For each of the levels mentioned above, investigators need to define realistic and achievable outcomes for a specific CST. The group favoured measurement of direct behavioural observation of the targeted communication skills either with simulated or real patients. For example, situational judgement tests where participants are asked how they would react in a given situation [146] could be an interesting way to measure the effect of CST.

#### Recommendations for future research

Based on the results from the systematic review and the discussion during the workshop, we make the following recommendations:

1. The choice of outcomes must be closely linked to the scope of communication training. Achieving a change in distal, generic outcomes requires the use of more intense interventions and larger evaluation studies compared to assessment of proximal, specific outcomes. Minimal clinically important differences should be defined beforehand.
2. Learning objectives must be adequately defined and targeted in the training. Proximal outcomes must be closely aligned with these objectives. Theoretical models or concepts of how these proximal outcomes will affect more distal outcomes should be made explicit.
3. Researchers should distinguish between three different levels for the evaluation of communication training: I) during the actual training process, II) during the interaction between patient and HCP, and III) after the interaction between patient and HCP. The intended impact of the training on these different settings and the respective proximal and distal outcomes should be explicitly defined, preferably derived from theoretical communication models.
4. Both experts and stakeholders, in particular patient representatives, should be involved in the definition of learning objectives, the development of the actual training, and the choice of outcomes.
5. A single outcome measure cannot cover all relevant outcomes to measure the effects of CST in oncology. Therefore, we recommend
  - Considering multiple potential outcomes. We suggest measuring the effects of communication training on all three domains identified: HCP, patient and interaction. Assessing the interaction is particularly relevant as concordance of judgements between patient and HCP should be investigated.
  - Avoiding measuring outcomes with known problems. For example, global ratings from patients on empathy or satisfaction have frequently exhibited ceiling effects and might be prone to social desirability.
  - Complementing quantitative assessments with qualitative assessments when possible as quantitative assessments seem unable to completely represent the communication process. These qualitative assessments could be an analysis of the communication encounter as well as qualitative interviews with CST participants or patients. Less common outcome measures, such as physiological stress reactions or situational judgement testing using case vignettes, might help to fill a gap.
  - Ensuring that the development and selection of outcome measures is transparent, clearly described and reproducible for other researchers as purpose-built outcome measures still have a central role in the evaluation of communication training programmes to reflect the content of the specific training.

## Discussion

This paper gives an overview of outcomes and the respective outcome measures previously used in the evaluation of communication training in oncology. It further discusses challenges experienced with outcome measurement in such studies and gives recommendations for future research. Many CSTs have been developed, implemented and evaluated to support health care professionals addressing specific communication challenges in cancer care. Our systematic review showed that outcomes and the respective outcome measures differ widely. The complementing workshop clearly described the challenges experienced in the evaluation of CSTs. To date, neither a specific outcome nor a specific outcome measure is a widely accepted standard tool. The large differences in content, extent and target populations of communication training in oncology can explain this. The lack of standardization, however, hampers building systematic and more conclusive evidence. Specific models of communication and theories how communication affects HCPs as well as patients in oncology can inform selection of appropriate outcomes.

An interesting finding is that outcomes and the respective outcome measures, as well as the challenges identified, are in most cases not specific to oncology. This suggests that generic communication processes can hardly be broken down to be disease specific. Exceptions are the outcome measures provided by the EORTC and the Cancer Research Campaign Workshop Evaluation Manual (CRCWEM), which have been specifically developed to assess the experiences of cancer patients.

The strengths of this study include its comprehensiveness as a descriptive review of outcome measures used in the evaluation of CSTs. The inclusion of systematic literature reviews on the effects of CSTs in this review revealed additional challenges, which are particularly relevant when study results need to be synthesized in meta-analysis.

Nonetheless, limitations of this work need to be taken into account when interpreting its findings. First, we could not register the review protocol, as its scope did not fulfil eligibility criteria for PROSPERO. Nonetheless, we wrote a protocol before we conducted the review. Furthermore, the syntheses of the specific outcomes in categories was difficult to standardize, since most primary studies made no specific distinction between outcome and the respective outcome measure. Although we grouped similar outcomes into categories, category borders are somewhat blurry, as often there are no clear definitions of the outcomes available. A further limitation is that a detailed analysis of psychometric quality in terms of reliability and validity of all outcome measures identified in the review has not been feasible. Hence, we cannot advise for or against the use of specific instruments, but we encourage the assessment of psychometric quality when one chooses an outcome measure. For this purpose, standardized tools can be used (e.g., EMPRO [147]). Another limitation is that the recruitment of the participants for the workshop did not follow a pre-specified protocol, but we selected potential participants based on their appearance as authors in the review as well as by recommendation by other participants. Furthermore, not all participants participated in the development of the recommendations after the workshop. Hence, these recommendations may not necessarily reflect the opinions of all participants in the workshop.

## Conclusion

Our review of the literature and the expert workshop made it clear that measuring the impact of CST in oncology is challenging. As human communication is complex, the heterogeneity of outcome assessment in studies is large. The complexity of the interventions and their potential effects hampers establishment of standard outcomes and outcome measures. Definition of a single core outcome suitable for each CST in oncology is unrealistic – there is a lack of consensus on what a core outcome could be and how it could be reliably assessed. Hence, we suggest a broad, reproducible assessment of communication on different levels derived from explicit learning objectives. Future research should emphasize the associations between these different perspectives on communication and develop theoretical frameworks that can guide the choice of relevant outcomes and meaningful effects of CSTs.

## Data Availability

Not applicable.
